# Violence in Healthcare: Legal Measures, Systemic Challenges, and Collective Accountability

**DOI:** 10.31729/jnma.8736

**Published:** 2024-08-31

**Authors:** Ashis Shrestha

**Affiliations:** 1 Journal of Nepal Medical Association, Pradarshani Marg, Nepal

## INTRODUCTION

There is growing concern about violence against healthcare workers, as it poses serious threats to the safety and well-being of people who devote their lives to delivering care. Healthcare workers are more susceptible to verbal and physical abuse at work, which has a significant negative impact on their morale and mental health. Over a prolonged period, the Nepal Medical Association (NMA) has consistently advocated for the protection of medical workers, emphasizing the urgent need for stringent laws and security measures. The NMA recently hosted awareness events on legal measures at 21 sites around the country to increase public awareness of the need to safeguard medical professionals and promote a safer workplace for all.

## LEGAL MEASURES

The Ordinance for the Safety and Security of Health Workers and Health Institutions (First Amendment) was enacted by the President of Nepal on May 8th, 2022. It was established under Article 114 (1) of the Constitution of Nepal.^1^ Section three of the amended act outlines the activities that are prohibited during medical treatment, while section 15 specifies the punishments for engaging in these prohibited activities. According to these two sections, if someone commits arson, vandalism, or any act that damages a health facility, they can be imprisoned for two to five years or fined between two to five lakh rupees, or both. This case will be handled by the district court. If someone beats or physically harms health workers or employees in a health institution or if the institution is locked or blocked, the offender can face up to three years in prison, a fine of up to three lakh rupees, or both. The district court will settle these cases. Similarly, if someone interferes with the treatment of patients or assaults health workers, threatens, abuses, or behaves indecently or insultingly, they can be jailed for up to one year or fined up to one lakh rupees, or both. These cases will be handled by the District Officer.^2^

## SYSTEMIC CHALLENGES

Strict laws are required to protect healthcare workers, but they are insufficient on their own.^3^ Violence against healthcare workers is a complex, multifaceted issue that is not adequately addressed by law.^4^ Though crucial, the application of legislation such as the Ordinance for the Safety and Security of Health Workers and Health Institutions is only one part of the whole. Addressing the causes of violence also requires institutional reforms within healthcare institutions, public awareness campaigns, education campaigns, and effective enforcement. Some of the main contributing causes to these accidents are high levels of stress, inadequate staffing, poor communication, and miscommu incidents going unreported.^5,6^ Similar incidents have been reported from neighboring countries like India,^7,8^ and China,^9^ where the relationship between doctors and patients has been considerably strained. Inadequate infrastructure for healthcare particularly in rural regions, exacerbates the issue in Nepal, resulting in long wait times and patient and family discontent.^10,11^ The increasing awareness among patients about their rights, coupled with unrealistic expectations of healthcare outcomes, has contributed to a volatile environment.^12^ Patients and their families frequently turn to violence when these expectations are not fulfilled, holding medical professionals personally responsible for institutional shortcomings. Overworked physicians who frequently cannot offer the quality of service they desire due to their limitations exacerbate this habit.

Poor communication is a common cause of violence against healthcare workers and is typically related to the excessive patient load that these staff must manage.^13^ This situation is not solely the fault of individual healthcare workers but rather a result of systemic failures. Poor communication is due to lack of time which is due to a limited number of healthcare workers trying to fulfill the expectations of too many patients. This unmatched healthcare worker-to-patient ratio is due to the limitation of vacant positions. This has a direct bearing on the Ministry of Health's funding allocations. Furthermore, the funding allocations are directly connected to the Ministry of Finance, Planning Commission, Province Assembly, and Federal Parliament.^14^ In the end, this cycle is linked to the nation's ability to generate income and the taxes it collects from its people. Poor communication is just a representative cause, several other causes follow the same vicious cycle of systemic challenges.

## COLLECTIVE ACCOUNTABILITY

Violence against healthcare professionals is therefore not only an isolated incidence but rather a manifestation of shortcomings in several tiers of administration and government. Responsibility for such incidents should not fall solely on the individual healthcare providers who are on the front lines delivering care. Rather, each link in this chain from government ministries and planning agencies to hospital administrators must shoulder their share of accountability. Moreover, healthcare is developed for disease and is run by healthcare workers. We need health care that is developed for people and is run by the health care system. Therefore, it is now time to shift the paradigm from quality healthcare service to an accountable quality healthcare system.
